# The application value of facial expression analysis system in violence risk assessment of patients with mental disorders

**DOI:** 10.1192/j.eurpsy.2025.604

**Published:** 2025-08-26

**Authors:** X. Ling, S. Wang, X. Zhou, N. Li, W. Cai, H. Li

**Affiliations:** 1 Shanghai Key Lab of Forensic Medicine, Key Lab of Forensic Science, Ministry of Justice, Shanghai Forensic Service Platform, Academy of Forensic Science; 2 Department of Forensic Medicine, School of Basic Medical Sciences, Fudan University; 3 The Smart Medicine and AI-based Radiology Technology Lab, School of Communication and Information Engineering, Shanghai University; 4 Shanghai Hongkou Mental Health Center, Mental Health Center Affiliated to Shanghai University School of Medicine, Shanghai, China

## Abstract

**Introduction:**

Patients with mental disorders often engage in extreme and unpredictable violent behaviors that seriously endanger the public security and stability of the society. Violence risk is commonly assessed by subjective judgement, which may lead to bias and uncertainty in the appraisal results. Existing expression recognition and analysis techniques have limitations in identifying the emotional states of patients with mental disorders.

**Objectives:**

The study aimed to explore the association between violent behaviors and facial expression in patients with mental disorders by machine learning algorithm, to evaluate the application value of facial expression analysis system in violence risk assessment of individuals with mental disorders.

**Methods:**

Thirty-nine patients with mental disorders were enrolled and assessed by using Modified Overt Aggression Scale (MOAS), positive and negative syndrome scale (PANSS) and brief psychiatric rating scale (BPRS). An emotional arousal paradigm was performed and the intensity of baisc emotions and expression action units was recorded before, during and after the paradigm. The processed quantitative data was used to generate one-dimensional waveform maps and two-dimensional time-frequency maps and then quantized feature data were extracted. A machine learning model with high accuracy was trained using these feature data, which can accurately determine the violence risk states of patients and output the probability. All individuals participated voluntarily and provided informed consent. This study was approved by the ethics committee of the Academy of Forensic Science.

**Results:**

The intensity difference of sadness, surprise and fear in different time periods was statistically significant. The intensity of the left medial eyebrow lift action unit was found significantly different before and after the emotional arousal. The intensity of anger and disgust was positively correlated with the MOAS scores, PANSS scores and BPRS scores. The features of time-frequency diagrams of 5 expression action units (medial eyebrow raise, eyebrow lowering, slightly open lips, chin drop and eye closure) and 8 basic emotions were selected and then support vector machine was used for triple classification, which is a classifier that can well distinguish the three stages of non-violence risk period, violence risk period, and post-violence risk period. In the 4:1 training-testing grouping, the classification accuracy reaches 91.2%.

**Conclusions:**

Featured expressive action units and various baisc emotions might be used to capture information associated with violent behaviors. The facial expression analysis system mentioned above can be used as an auxiliary tool to assess the potential risk of violence in patients with mental disorders.

**Disclosure of Interest:**

X. Ling: None Declared, S. Wang: None Declared, X. Zhou: None Declared, N. Li: None Declared, W. Cai: None Declared, H. Li Grant / Research support from: This study was supported by National Key R & D Program of China [grant number 2022YFC3302001], National Natural Science Foundation of China [grant number 81801881], Science and Technology Committee of Shanghai Municipality [grant numbers 20DZ1200300, 21DZ2270800, 19DZ2292700].

